# Introduction: Mentholated cigarettes and public health

**DOI:** 10.1186/1617-9625-9-S1-I1

**Published:** 2011-05-23

**Authors:** Allison C Hoffman

**Affiliations:** 1 Center for Tobacco Products, Food and Drug Administration

## 

The Food and Drug Administration’s Center for Tobacco Products (CTP) oversees the implementation of the Family Smoking Prevention and Tobacco Control Act, H.R. 1256. Among its many provisions, section 907(a) of the Tobacco Control Act banned the use of characterizing flavors, specifically excluding only tobacco and menthol flavors from the ban. According to section 907(e)(2), “[n]ot later than 1 year after its establishment, the Tobacco Product Scientific Advisory Committee shall submit to the Secretary the report and recommendations required pursuant to [menthol],” which, according to section 907(e)(1), is “the issue of the impact of the use of menthol in cigarettes on the public health, including such use among children, African-Americans, Hispanics, and other racial and ethnic minorities.” The inaugural meeting of the Food and Drug Administration’s Tobacco Products Scientific Advisory Committee (TPSAC) was held March 30–31, 2010 in Washington, DC. In preparation for this meeting, CTP reviewed peer-reviewed literature on menthol and tobacco as indentified by the National Cancer Institute’s “Bibliography of literature on menthol and tobacco”, and seven scientific presentations were made, including the use of menthol cigarettes (epidemiology), smoking topography and sensations associated with menthol cigarettes, marketing of menthol cigarettes, menthol cigarettes and initiation of smoking behavior, menthol cigarettes and nicotine dependence, menthol cigarettes and cessation of tobacco use, and the health effects of menthol cigarettes as compared to non-menthol cigarettes.

Following this initial presentation, additional information was collected through FDA investigation, as well as recommendations from the public, research community and tobacco industry, resulting in comprehensive reviews. Thus, this supplement brings together the extant peer-reviewed information on these seven topics.

## Competing interests

The author declares that they have no competing interests.

